# 

**Published:** 1866-02

**Authors:** 


### CONTENTS.

ORIGINAL ESSAYS AND COMMUNICATIONS.

Dental Materia Medica ........ 55
Therapeutic Effects of Mercury in Acute Periostitis ........ 58
Cold Applications to Aching Teeth ........ 62
Miscellaneous Items ........ 64
Diagnosis ........ 66
Sensitive Dentine in a Pulpless Tooth ........ 67
Severe Affection of an Eye resulting from a Diseased Tooth ........ 69

SELECTIONS.

The Hypophosphites—Their Therapeutic Value ........ 71
Chloroform—Its Action and Administration ........ 74
India Rubber Cones for Polishing Vulcanized Rubber ........ 77
Re-amputation for Intense Neuralgia in a Stump. Anæsthesia induced by Nitrons-Oxide Gas ........ 79
Anæsthesia from the Internal Use of Chloroform ........ 80
On the Germinal Matter of the Blood, with Remarks upon the Formation of Fibrin ........ 81
Injection of Air into Abscesses and Fistulæ ........ 82
Note Taking ........ 83
The Rattlesnake Poison ........ 83
Re-production of Bone ........ 84
Nitrous Oxide Gas in Surgical Operations ........ 85
Dissecting Abscesses of the Leg ........ 85
Monograph on Glycerin and its Uses ........ 86
Nitrous Oxide Gas in Capital Operations ........ 86
Nitrous Oxide ........ 88

CORRESPONDENCE

About the Editor of the Cosmos, etc ........ 89
Foreign Correspondence ........ 94
Letter from C. W. Spalding ........ 96

EDITORIAL.

Times ........ 98
Examination for Degrees ........ 99
Colorless Iodine ........ 100
Resolution of the Mad River Valley Association ........ 100

